# Informal knowledge transfer in the period before formal health education programmes: case studies of mass media coverage of HIV and SIDS in England and Wales

**DOI:** 10.1186/1471-2458-7-293

**Published:** 2007-10-17

**Authors:** Nick Hilliard, Rebeka Jenkins, Nora Pashayan, John Powles

**Affiliations:** 1 University of Cambridge School of Clinical Medicine, Addenbrooke's Hospital, Hills Road, Cambridge, CB2 2SP, UK; 2 Department of Public Health and Primary Care, Institute of Public Health, University Forvie Site, Robinson Way, Cambridge CB2 0SR, UK

## Abstract

**Background:**

How advances in knowledge lead via behaviour change to better health is not well understood. Here we report two case studies: a rapid reduction in HIV transmission in homosexual men and a decline in Sudden Infant Death Syndrome (SIDS) that took place in the period before the relevant national education programmes commenced, respectively, in 1986 and 1991. The role of newspapers in transferring knowledge relevant to reducing the risk of AIDS and SIDS is assessed.

**Methods:**

HIV

Searches were made of *The Times *(1981–1985), *Gay News *(1981–1984) and, for the key period of April to June 1983, of eight newspapers with the highest readership. Information on transmission route and educational messages were abstracted and analysed.

SIDS

Searches were made of *The Times *and the *Guardian *(1985–1991), *The Sun *(selected periods only, 1988–1991) and selected nursing journals published in England and Wales. Information on sleeping position and educational messages were abstracted and analysed.

**Results:**

HIV

Forty-five out of 50 articles identified in newspapers described homosexuals as an at risk group. Sexual transmission of AIDS was, however, covered poorly, with only 7 (14%) articles referring explicitly to sexual transmission. Only seven articles (14%) associated risk with promiscuity. None of the articles were specific about changes in behaviour that could be expected to reduce risk. Gay periodicals did not include specific advice on reducing the number of partners until early 1984.

SIDS

Out of 165 relevant articles in *The Times *and 84 in the *Guardian*, 7 were published before 1991 and associated risk with sleeping position. The reviewed nursing journals reflected a pervasive sense of uncertainty about the link between SIDS and sleeping position.

**Conclusion:**

Presumptively receptive audiences responded rapidly to new knowledge on how changes in personal behaviour might reduce risk, even though the 'signals' were not strong and were transmitted, at least partly, through informal and 'horizontal' channels. Advances in knowledge with the potential to prevent disease by behaviour change may thus yield substantial health benefits even without the mediation of formal education campaigns ('interventions'). Formal campaigns, when they came, did make important additional contributions, especially in the case of SIDS.

## Background

Although the advance of knowledge is the most important distal (or 'wider') determinant of health improvement, the means by which new knowledge produces its beneficial effects on health are often far from clear. This is especially true when these beneficial effects depend on informed behaviour change by the public. Here we consider changes away from two high risk behaviours that took place in Britain, mainly during the 1980s – movements away from sexual practices associated with human immunodeficiency virus (HIV) transmission and away from putting babies to sleep on their fronts, a practice now known to increase risk of sudden infant death (SIDS).

One interpretation of how the advance of knowledge leads via behaviour change to better health emphasises the formal 'top down' pathway. In this view, key experts and politicians respond to new knowledge by building a consensus on what needs to be done to inform the public of the new knoweldge. Formal health education campaigns are then mounted. In the case of sleeping position and SIDS, 'top-down' sequences of this kind have been documented across several European countries by McKee and others [1]. Such interpretations emphasise the role of the formal education programmes instituted from above (i.e. 'interventions') in putting the new knowledge to work for preventive purposes.

**Figure 1:**
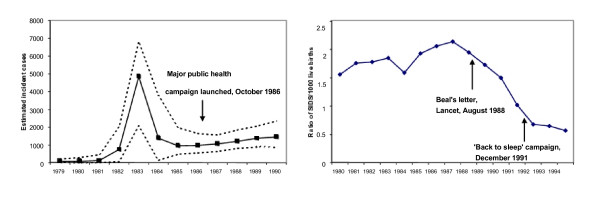
a) Estimated incidence and upper and lower 95% uncertainty range (Bayesian methods) of HIV infection in homosexual and bisexual men, England and Wales, 1979–1990; b) Ratios of deaths attributed to SIDS per 1000 live births, England and Wales, 1981–1994. 1a) Sources: Incidence estimates [35], timing of campaign [36]. 1b) Sources: Deaths [OPCS], timing of 'Back to sleep' [December 1991].

A major difficulty with this 'top down' interpretation is that in many instances it has been discovered (retrospectively) that substantial change away from high risk behaviours occurred before the formal health education campaigns actually began. This is the case for the two examples from England and Wales which we explore here: a rapid reduction in HIV transmission in homosexual and bisexual men which took place in the period before the national education programme commenced in 1986 (Figure 1a) and the decline in SIDS mortality which began well before the start of the national education programme in 1991 (Figure 1b). Coincidentally or not, the SIDS decline began in 1988, the year in which the epidemiological evidence on the association between sleeping position and risk was first formally synthesised, in a letter to the Lancet [2]. (Further background on the evolution of aetiological knowledge for these 2 conditions is given in Additional file 1. doc, 'Additional historical background'; Additional file 2. doc, 'Additional Table 1: Brief chronology of scientific discoveries and publications on HIV/AIDS'; Additional file 3. doc, 'Additional Table 2: Chronology of scientific publications on the association between SIDS and sleeping position').

We set out to assess the role of print media in transmitting knowledge about how the risk of these two conditions could be reduced – based in part, on earlier studies suggesting an important role for newspapers and magazines in the increase in smoking cessation in the US during the 1950s and 60s [3].

### Initial and amended research strategy

Our initial hypothesis had been that UK newspapers provided information that enabled the relevant target populations to reduce the risk of, respectively, AIDS and SIDS. We soon discovered that the stories were more complicated in each case.

With AIDS, for example, Nicoll et al (2001) [4] had already noted that newspaper coverage of sexual practices as risk factors for AIDS had been relatively limited during the critical period of 1983 – 1984. These authors consequently invoked a putative 'campaign' within the homosexual population as the hypothetical vehicle for transmitting knowledge on how to stop the spread of HIV.

In the case of SIDS in Britain, Scott et al in 1993 [5] had also doubted whether newspaper coverage of sleeping position as a risk factor had been sufficient to explain the reduction in mortality. In its place they invoked a vanguard role for prescient health visitors conveying the new knowledge ahead of the change in official recommendations.

In the light of these earlier observations we now sought to ensure that our methods were sufficiently sensitive to capture all potentially relevant communications. Alternative explanations of why behaviour had changed had also to be considered, including the two just noted – a 'campaign' within the homosexual population (in the cases of HIV) and early advice from health visitors (in the case of SIDS).

## Methods

### HIV

#### Newspapers and Television

National newspapers with the highest readership were identified [6] as *The Times*, *Daily Telegraph, Guardian, Sun, Daily Mail, Mirror, The Sunday Times*, and *Mail on Sunday *and were selected for this study. *The Times *was searched from 1981 to 1985 using The Times Digital Archive 1785–1985 [7]. This identified the period of April to June 1983 as one of increased media attention. The rest of the newspapers were therefore searched manually for this period looking for any content which could, plausibly, have contributed to behavioural change. Newspaper searching identified the broadcast of a BBC TV Horizon documentary *'Killer in the village' *on 25^th ^April 1983 as a significant event and its transcript was retrieved and reviewed [8].

#### Gay periodicals

*Gay News*, a national gay newspaper of the time, was searched from 1981–1984.

#### Data abstraction

All articles providing news about AIDS, HIV in blood products, commentary about AIDS, or having a message that could plausibly influence behaviour were included. Data were abstracted from each article using a data abstraction form which included fields for the source of the information reported, any risk groups identified, transmission routes discussed and any identification of changes likely to lower risk (Additional file 4. doc, 'Additional Table 3: Data abstraction form for HIV/AIDS articles).

### SIDS

#### Newspapers

The review of newspapers covered the period from 1985, before decline in SIDS, to December 1991, when the national media campaign was launched.

Three national newspapers were selected for study; *The Times *and *The Sunday Times *(London), *Guardian *and *The Sun*. *The Times *and the *Guardian *were chosen as the two indexed UK newspapers covered by the Lexis Nexis electronic database over the time-period of interest. Various search terms were tested and the combination ["sudden infant death" OR "cot death"] was found to be sufficient to locate all articles mentioning the subject of SIDS.

*The Sun *was chosen as the newspaper with one of the highest circulation at the time of interest. A hand search was directed towards key times of likely media interest over 1988–1991, as determined by publications in the scientific literature and articles in *The Times *and *Guardian *newspapers.

#### Nursing Journals

A Pubmed search was performed using the search term "sudden infant death [MESH]" and the limits "Languages – English", "Subsets: Journal Groups – Nursing" and "Published in the last – 1.1.1985–31.12.1992". Articles from 1992 were included here to gauge the response to the national campaign as reported in the nursing journals.

#### Data abstraction

All articles mentioning SIDS were reviewed. Articles reporting on sleeping position were further examined for whether sleeping position was a major topic, the framing of the topic, the main themes and any information about how risk might be reduced. Data were abstracted from each article using the data abstraction form (Additional file 5. doc, 'Additional Table 4: Data abstraction form for SIDS articles').

### Analysis

Articles on HIV and SIDS were reviewed by two of the authors respectively (NH, RJ). When there was uncertainty about the content of the articles, all the authors were contacted for clarification. To answer the study question it was sufficient to examine the articles for knowledge content.

## Results

### HIV

We identified 288 articles in *The Times *from 1^st ^January 1981 – 31^st ^December 1985, of which only three were published before April 1983. From April to June 1983, 50 relevant articles were found in the 8 national newspapers. Of these three appeared on the front page. The majority of the articles 35 (70%) were in the news section, 7 (14%) were commentaries, 2 (4%) were health reports and 6 (12%) were brief summaries of AIDS related television programmes. The coverage appeared in broadsheets (higher quality newspapers) in 32 (64%), and only 10 (20%) of the articles directly referred to research reports. References to named or anonymous 'official' and 'expert' sources were made in 19 (38%) articles.

#### General newspapers and Television (April to June 1983)

Uncertainty and fear over the future course of the AIDS epidemic, or unclear aspects of the disease featured directly in 38 (76%) of the articles. Homosexuals were identified as a risk group in 45 (90%) articles. Sexual transmission of AIDS was however covered poorly, with only 7 (14%) articles referring explicitly to sexual transmission, of which one article referred to anal sex. Blood, or blood products, as a mode of transmission featured in 25 (50%) articles (Table 1).

**Table 1:** Mentions of AIDS with illustrative quotations, classified by major themes, selected UK newspapers, 1981–1985

**Early coverage**
"AIDS contagious and spreads sexually" [13]
"Latest research does indicate that AID could very possibly be spread through a virus, which makes it a sexually transmitted disease" [14]
**Uncertainty and fear over the future course of AIDS epidemic**
"Now spread to other groups, fears may reach to London" [37]
"Inexorable extension into general population" [38]
"Serious fear of spreading abroad [from US]" [39]
"Death of victims slow and terrible" [39]
"Fears that the true number of cases of AIDS in Britain may be much higher" [40]
"First hard evidence that...contracted by non-homosexuals" [41]
"Fear may jump to straight population" [42]
"[on the gay community] feeling of isolation and separation" [42]
Headline: "Gay plague kills gran" [43]
"Terrified of contact with sufferers [Headline: Gay sex bug victims are lepers] " [44]

**Transmission route**
"First thought to be only sexually transmitted" [45]
"Sexually transmissible killer disease" [46]
"Transmissible agent in blood or semen" [47]

**Risky behaviours identified**
"[homosexuals] vulnerable and promiscuous population" [38]
"[referring to less cases in the UK than the USA] less extreme promiscuity and little multiple drug use, both believed to be factors in spread" [42]
"Promiscuity as...revolutionary sexual brotherhood...genuine fears for health put on defensive" [42]
"Chastity may become latest fashion" [48]
"Indiscriminate promiscuity" [39]
"Take fewer partners, but not necessarily have less sex" [16]

The role of promiscuity in the epidemic's spread was described in 7(14%) articles, with 6 (12%) of these giving some abstract message that number of partners should be reduced. None of the identified articles gave any other advice on safe sex measures.

The Horizon documentary 'Killer in the Village' [8] generated interest in the media and marked the beginning of extensive coverage of AIDS in the mass media and the increase in calls to Gay Switchboard [9]. This documentary presented AIDS as a condition caused by a transmissible agent, present in blood with promiscuous homosexual men being at especially high risk.

#### Gay media and educational activities within the gay community

We did not find evidence consistent with an organised 'campaign' among the gay community during the period from February 1981 to February 1984. There were only 20 published articles and letters related to AIDS in the *Gay News *and these turned out to be diffident in identifying specific behaviours associated with risk. Promiscuity was mentioned as a behavioural risk factor, with some uncertainty, as early as 12^th ^November 1981, with an article entitled "Gay cancer or mass media scare?" [10]. This article underplayed theories on sexual transmission and minimised the disease as a solely gay one. The confusion over the role of sexual activity as a risk factor continued throughout 1982 with a report on 27^th ^May [11] of an Atlanta enquiry that tried to minimise the sexual aspect of the disease, while stating that heterosexuals can catch it too. An article on 22^nd ^July 1982 [12] suggested that 'poppers' (amyl nitrate ampoules) may be the cause but also stated that "promiscuity is an important consideration".

Definite recognition that AIDS is "contagious and spreads sexually" finally appeared on 2^nd ^September 1982 [13]. The earliest specific alert that British gay men received was in a letter written by an American tourist published as the main front page article in '*Capital Gay*' on 3^rd ^September 1982 [14]. It contained the first reference to British AIDS cases and to the possibility that it was a viral disease that could be sexually transmitted:

"On recent travels through Europe I have noticed how terribly unaware most European gay men are of the recent AID (Acquired Immune Deficiency) epidemic outbreak in the USA. Approximately two new cases are reported every day... Latest research does indicate that AID could very possibly be spread through a virus, which makes it a sexually transmitted disease. Perhaps gay men in Europe can learn from the terrible news we are hearing here in the US and possibly prevent a serious spreading of the disease into the European communities. Please do whatever you can to inform and alert your readers."

Further reporting of AIDS in *Gay News *through 1983 was limited due to a period of insolvency of the publication from March till August 1983. After resumption there were few reports from the gay community on AIDS transmission, aside from an article on 18^th ^August 1983 [15] warning against sexual contact with American visitors at the Edinburgh festival.

The first unequivocal and specific advice printed in *Gay News *came in February 1984 [16], when it quoted a doctor: "take fewer partners, but not necessarily have less sex".

Other educational activity within the gay community included a seminar in Conway Hall on 21^st ^May 1983 attended by about 200 people. The Terrence Higgins Trust was first set-up as a fundraising organisation, aiming to finance research into AIDS. It took on an educational role from 1984. The Gay Medical Association set up an AIDS working-group and began its educational work by drafting a leaflet for General Parishioners [14].

### SIDS

#### The Times and Guardian

**Figure 2:**
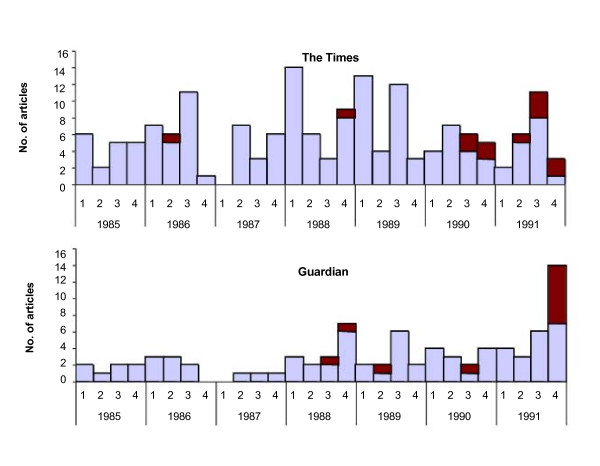
Articles mentioning SIDS with (darker colour) and without (lighter colour) mention of sleeping position, *The Times *and *Guardian*, quarterly counts for January 1985 to December 1991.

We found 165 articles in *The Times *and 84 in the *Guardian *on SIDS between January 1985 and December 1991. Of these, just 20 (8%) mentioned infant sleeping position, of which only seven were published before 1991 (Figure 2). In 13 of these 20 articles, sleeping position was the main topic with 5 mentioning the issue in the headline. Twelve mentioned studies from the scientific literature and 7 quoted "experts" directly. Three highlighted the fact that opinion was divided (Table 2).

**Table 2:** Mentions of SIDS with illustrative quotations, classified by major themes, selected UK newspapers, and nursing journals, 1985–1992

**Divided opinion among professionals and confusion of parents**
"Professor John Emery...has written to the Lancet questioning the evidence for the supposition that the position the baby sleeps in is an important factor" [49]
"Dr Peter Fleming, a consultant paediatrician at the Royal Hospital for Sick Children in Bristol and a member of the foundation's scientific advisory board, yesterday strongly criticised Acheson for issuing advice to parents to put babies on their sides" [50]
"The change of advice [about sleeping position] has created anxiety and uncertainty among some parents and professionals" [50]
A mother was quoted "Simply following the latest theory on [SIDS] prevention may not be as simple as it sounds. [With] my first child...I felt moderately secure that I was doing the right thing by carefully placing him [as] advised to do in hospital...Now we learn that there are other views" [50]
**Association between prone sleeping position and SIDS risk**
"Low rate of SIDS in Hong Kong may be related to the Chinese practice of placing the sleeping infants on their backs" [17]
"Recent reports in the BMJ [51,52] suggest that the position in which a baby sleeps may have a lot to do with why healthy babies unexpectedly die in their cots" [18]
"Most of those who died were sleeping in the prone position" [19]
"There are grounds for thinking that the prone position may contribute to cot death" [53]
"Reflecting recent concerns that putting babes on their stomachs may contribute to cot deaths" [54]

**Uncertainty within the nursing profession**
"I think this latest advice is an anathema to many health professionals, and we have had some quite irate phone calls from some of our members" [24]
"...recently there has been much discussion about whether babies placed in the prone position rather than supine are more at risk of cot death. Although a few studies found that more babies were found lying in a prone position and this could be a contributory factor, this has not been statistically proven" [21]
"There are conflicting opinions. Unless there is an obvious reason for the prone position no 'right' position emerges" [23]
"A non-prone position does not guarantee not to have SIDS. However in response to these findings [review of literature from 1988] the Department of Health has published and widely circulated a leaflet advocating a non-prone position. The effect of this campaign has yet to be seen" [55]
"I have picked up some information about the new position casually from the media but since the birth neither the GP, midwife or health visitor have raised the subject voluntarily...In fact, I felt that maybe [the health visitor] wasn't so convinced herself" [56]
"...many nurses trained since the 1960s are finding the change hard" and the Alison Stewart, the Avon infant mortality study coordinator states "the government have given health professionals little background evidence to reinforce the programme, and until they become convinced that the position is safe we are dramatically reducing the effectiveness of our message" [56]

A possible association between the prone sleeping position and SIDS risk was discussed in three different ways. Six articles merely stated that a link between SIDS and sleeping position had been suggested. Some examples include; [the] "low rate of SIDS in Hong Kong may be related to the Chinese practice of placing the sleeping infants on their backs [17], [a] "recent report in the BMJ suggests that the position in which a baby sleeps may have a lot to do with why healthy babies unexpectedly die in their cots"[18] (Table 2). Three articles reported that a higher proportion of SIDS infants are found prone, for example: "most of those who died wore more clothing and bedding and were sleeping in the prone position"[19]. Only 5 go so far as to state explicitly that babies that sleep prone have an increased risk of SIDS and none of these appeared before June 1991. Sixteen articles listed multiple factors, including other aspects of infant care that had a potential role in SIDS.

#### The Sun

Over seven selected months from 1988 to 1991 (chosen from the distribution of stories in *The Times *and the *Guardian*), there were 18 articles on SIDS, of which only two referred to sleeping position and both of those appeared after June 1991.

Most were framed as 'human interest' stories; referring, for example, to losses by well known persons. Most notably there was no reference to sleeping position in a doctor's column entitled "How to cut the risk of cot death"[20], which did discuss temperature, bed-clothes, breast and formula feeding, smoking and the symptoms of respiratory illness.

#### Nursing Journals

**Figure 3:**
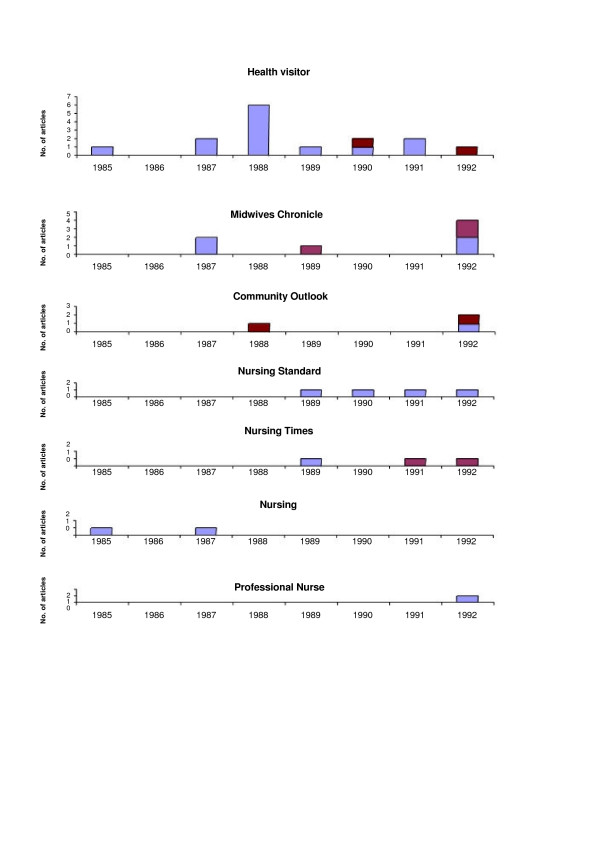
Articles mentioning SIDS with (darker colour) and without (lighter colour) mention of sleeping position, nursing journals, England and Wales, yearly counts for 1985 to 1992.

From January, 1985 to December 1992, we found 37 articles on SIDS published in 7 English nursing journals. We only found four articles published before 1992 that mentioned sleeping position (Figure 3). These four articles reflected not so much the strength of epidemiological evidence as a sense of uncertainty within the nursing profession about how such evidence was to be interpreted. "...although a few studies found that more babies were found lying in a prone position and this could be a contributory factor, this has not been statistically proven" [21]. In June 1989 a correspondent to the *Midwives Chronicle *drew the attention of her colleagues to "recent publications" and posed the question: "can we reduce numbers of SIDS by educating our clients? Should we recommend a non-prone sleeping position" [22]. In the *Nursing Times*, (April 1991), an author concedes that "advice given to parents is important. The evidence would suggest placing babies on their back or sides reduces their risk of SIDS" however they also note "Unless there is an obvious reason for the prone position no 'right' position emerges. There are conflicting opinions" [23].

Even after 1992, there were doubts about the recommended sleeping position among health visitors (Table 2). Margaret Buttigieg, head of professional services for the Health Visitors' Association, was quoted in *The Times *[24] "I think this latest advice [on sleeping position] is an anathema to many health professionals, and we have had some quite irate phone calls from some of our members".

## Discussion

We consider it unlikely that we have selectively missed articles that gave more forthright interpretations of evidence either on sleeping position and SIDS or on the sexual transmissibility of HIV. Of the 77 items found by Weller et al (1984) [9] on HIV/AIDS across all national media, 50 were identified from the 8 sources used in this study. Our search for articles on SIDS in *The Sun *were admittedly incomplete as coverage in the *Sun *did not follow a similar pattern to *The Times *or *Guardian*. Consequently, it was difficult to predict when *The Sun *would run a succession of articles about the same event (for example, in January 1989, 4 of the 8 articles referred to the death of a baby who had starred on television, no mention of which was made in either of the broadsheets). There is, however, no reason to believe that *The Sun*, with its focus on human interest stories, would have reported in articles we missed on scientific debate and come to a more definite conclusion than *The Times *or *Guardian*. Therefore, it is highly unlikely that a major impetus for the change in parenting behaviour was coming from tabloid newspapers such as the *The Sun*.

The only plausible explanation for the falls in HIV incidence and SIDS deaths before the national campaigns is that relevant risk determining behaviours changed favourably. There is supporting evidence for such changes.

The fall in the incidence of HIV in homosexual men occurred contemporaneously with change in reported sexual behaviour [25]. Weber et al (1986) [26] showed a marked reduction in number of partners per year between 1982 and 1985 in homosexual men recruited through STD clinics in London.

If behaviour did change, what new information provoked the change? Our prior hypothesis that clear and strong messages had passed through the general and professional media was not upheld. Our findings suggest a more subtle role for the print media, one that acts in combination with informal and 'horizontal' communication in the at risk population. Faced with a similar explanatory dilemma, Nicoll et al [4] asserted that there had been a 'campaign' to spread knowledge in the homosexual community during the early period of limited and equivocal newspaper coverage. However we have failed to find evidence of activities sufficiently coordinated and formal to be described as a 'campaign' and are therefore led to suspect the importance of less formal processes.

Berridge has noted how the initial responses at the clinical and scientific level as well as among gay men were essentially of a self-helping and voluntarist nature [27]. Some gay men were regular travellers to the US and began to hear of people dying of 'strange cancers'. According to Berridge, travellers acted as 'missionaries' bringing back such knowledge as existed [27].

The news of the arrival of the first AIDS patient to St Mary's Hospital, London, in 1983 was published in the gay press. The BBC Horizon programme, *Killer in the village *soon followed and portrayed graphically what the UK gay community could expect. According to *Capital Gay*, this became the 'main topic of discussion in every pub and club and over dinner tables throughout London' [27].

During this period, the organised gay response to AIDS (such as the Terrence Higgins Trust and the Gay Medical Association) began to heighten and also significantly to change focus at the same time. Consequently, horizontal communication as well as the vertical communication could have raised awareness and helped foster changes in sexual behaviour. Alongside these activities there was the tangible reality of deaths among gay men. These two factors could have contributed to the changes in behaviour that materially reduced HIV infection rates.

In the case of SIDS, newspaper reporting in the period before the formal 'Back to sleep' education campaign, is notable for its equivocation on the importance of sleeping position. Only a small proportion of the identified articles mentioned a link between SIDS and sleeping position. The nursing journals we searched reflected a pervasive sense of uncertainty within the nursing profession about this link and we found no evidence to support the hypothesis that health visitors acted as a vanguard offering advice on sleeping position in advance of the change in official policy.

Despite the uncertain and conflicting messages in the general media and the nursing journals, deaths attributed to SIDS fell steadily from 1988. There is evidence of concurrent changes in parental behaviour. A retrospective study in East Anglia found that the proportion of babies exposed to the hazardous front sleeping position roughly halved between 1989 and 1990 – from 37% to 19% [28] and Gilbert et al [29] showed that the proportion of babies sleeping prone started to decline before 1990. Similar findings have been reported from New Zealand. Changes in parental behaviour were apparent from the sleeping positions of controls in a national case/control study. There was a concurrent fall in SIDS. Both these changes happened ahead of the formal education campaign [30]. In Scotland, the rate of SIDS also fell in advance of the national education campaign [31].

The equivocal and limited coverage of sleeping position by the newspapers, during this period, leaves the source of such a change mostly unexplained. Nor is there evidence of widespread prescience on the part of primary care professionals – health visitors and general practitioners – at least in England. There was a widespread failure to appreciate the import of epidemiologic findings – at least until their application in national policy coincided with dramatic falls, as in New Zealand [32]. Leading UK experts were reportedly convinced by the New Zealand experience reported at the founding conference of the European Society for the Study and Prevention of Infant Death held in Rouen on June 6–7, 1991 [32].

We are therefore led again to consider additional less formal processes to explain trends before 1991. Parents may have been particularly receptive just to evidence that professionals were uncertain about how babies should be put to sleep and to a subtle shift in newspaper portrayals of SIDS over this period – from unexplained family tragedies to potentially preventable events for which an increasing number of risk factors were being investigated. A shift of emphasis towards potential preventability and a listing of sleeping position as one factor under investigation may have helped induce a proportion to revert to the lay practices that preceded the official support for the prone position during the 1960s and 1970s. It is possible that grandmothers passed on conserved knowledge of prior practice. This may have been assisted by informal processes serving to amplify the otherwise weak messages in the public domain about relatively greater safety of putting the babies on their back- for example mother and baby discussion groups and discussions among and with the minority of primary care professionals who were earlier responders to the developing evidence. These explanations are admittedly speculative but we lack credible alternatives.

Knowledge about the sexual transmissibility of HIV and about the risks to babies when they are put to sleep on their fronts yielded its benefits to health when it was acted on by the relevant target populations. The question of how the knowledge actually reached the relevant actors in the periods before official national campaigns is of considerable intrinsic interest and potentially wider relevance. Our evidence suggests that simply invoking the role of the print media is insufficient. Although it can only be indirectly supported, the conclusion seems inescapable that complex informal processes of knowledge transfer and 'innovation diffusion' [33] have also played a central role.

## Conclusion

Health promotion specialists are often frustrated by difficulty of demonstrating effects of their educational efforts. These two case studies show that under some circumstances 'receptive' audiences may respond rapidly to new information – even if it requires them to discriminate carefully between weak and unclear signals coming through both 'vertical' and 'horizontal' channels. Health improvement through behaviour change need not always depend on 'interventions' by professionals and public health officials. Given a presumptively strong interest in risk avoidance and means of risk reduction that are within the competence of the target group, the advance of knowledge may almost be enough in itself to yield substantial health gains – needing only to be supplemented by routine mass communications and the normal repertoires of social interaction. Formal programmes often come after some delay and may extend and enhance the baseline gains. Assessments of societal gains from advances in medical knowledge that limit themselves to the contributions of formal 'interventions' will, in cases like those explored here, seriously underestimate the full benefits [34].

## Competing interests

The author(s) declare that they have no competing interests.

## Authors' contributions

NH and RJ devised the data abstraction forms and reviewed the newspapers and research articles. NP supported in literature search and interpretation. JP conceptualised the project and supported in literature search and interpretation. All authors contributed to the writing of the manuscript.

## Pre-publication history

The pre-publication history for this paper can be accessed here:



## Supplementary Material

Additional file 1 Additional historical background. Further historical background on the evolution of mortality from the causes of interest and on the development of scientific knowledge relevant to the prevention of the two conditions of interest. Click here for file

Additional file 2 Additional Table 1: Brief chronology of scientific discoveries and publications in HIV/AIDS. From first description of the syndrome in March, 1981 to the licencing of blood tests in 1985. Click here for file

Additional file 3 Additional Table 2: Chronology of scientific publications on the association between SIDS and sleeping position. Reports of epidemiological studies of SIDS from 1965 to 1991. Click here for file

Additional file 4 Additional Table 3: Data abstraction form for HIV/AIDS articles. Data abstraction form for HIV/AIDS articles. Click here for file

Additional file 5 Additional Table 4: Data abstraction form for SIDS articles. Data abstraction form for SIDS articles. Click here for file
