# Supplementary material for: Informal knowledge transfer in the period before formal health education programmes: case studies of mass media coverage of HIV and SIDS in England and Wales
Source: BMC Public Health. 2007 Oct 17;7:293. doi: 10.1186/1471-2458-7-293 (PMC2194775; doi:10.1186/1471-2458-7-293)
Supplement: Additional file 1 — Additional historical background. Further historical background on the evolution of mortality from the causes of interest and on the development of scientific knowledge relevant to the prevention of the two conditions of interest. [file 1471-2458-7-293-S1.doc]

### Additional text: Further Historical Background

##### HIV in England and Wales

Figure 1 shows the central estimates and the 95% uncertainty intervals for HIV incidence in homosexual and bisexual men in England and Wales, prepared by back-projection from the subsequent AIDS epidemic. HIV incidence rose sharply to a peak in 1983 and fell back equally sharply to around one quarter of its peak levels by mid 1985 [1]. However, the first national education campaign, ‘Don’t aid AIDS’ did not begin until March 1986 with a series of national newspaper advertisements [1,2].

##### Evolution of knowledge of the causes of AIDS

This newly manifest disease was named aquired immune deficiency syndrome (AIDS) by US authorities in August 1982. By 1983 epidemiological and microbiological researchers in the US were convinced that it was caused by an unidentified agent transmitted via sexual contact, blood or blood products [3]. The chronology of discovery is set out in Additional file1.1. doc, ‘Additional Table 1: Brief chronology of scientific discoveries and publications in HIV/AIDS’.

Changes in sexual behaviour appear to have begun promptly. US studies report reductions in numbers of partners from 1982 [4]. For London, Weller et al [5] reported a reduction in gonorrhea in homosexual men in the second and third quarters of 1983. This was described as concurrent with an increase in media coverage of aquired immunodeficiency syndrome (AIDS).

#### SIDS

##### Attributed deaths

There are interesting parallels in the story for SIDS. Figure 1b shows the trend between 1980 and 1995 in deaths attributed to SIDS in England and Wales. From the peak value of 2.14 deaths per 1000 live births in 1987 there was a fall of over 50% to the figure of 1.02 per 1000 live births for 1991. The official government programme advocating ‘Back to sleep’ began on December 11, 1991 [6].

##### Putting babies to sleep on their fronts and SIDS

The chronology of scientific publications is set out in Additional file 1.2.doc, ‘Additional Table 2: Chronology of scientific publications on the association between SIDS and sleeping position’.

Professional opinion in favour of putting babies to sleep on their fronts (i.e. prone) dates from mid 1950s when this became the recommended position for the care of premature babies in hospital, a recommendation that was subsequently generalised to all babies [7]. Doubts about the safety of the prone position were raised after the ecologic observation in 1985 that Chinese infants in Hong Kong seemed rarely to die of SIDS and were traditionally put to sleep on their backs [8].

In August 1988, Beal showed, in a letter to the Lancet, that when the results of nine case-control studies were formally combined, the association of the prone sleeping position with SIDS risk was highly significant (p < 0.01), even though only some of the primary study reports had emphasised this association [9].

Gilbert et al were to show much later (in 2005) that if (counterfactually) modern methods of evidence synthesis had been available at the time, the data published by 1970 would have been sufficient to demonstrate a significant association between the prone sleeping position and SIDS risk (pooled OR 2.93, 95% CI 1.15, 7.47) [7].

In the Netherlands, official recommendations on how babies should be put to sleep changed early following discussion of results from an epidemiological study in 1987 [10]. Experts who were influential in Britain appear to have changed their opinion rather later, in response to the presentation of results from epidemiological studies in New Zealand, The Netherlands and Bristol of which they became aware at the founding conference of the European Society for the Study and Prevention of Infant Deaths (now ISPID) (held in Rouen, 6-7^th^ June, 1991) [11]. In the US, official advice on sleeping position was slow to change because paediatricians there gave more credence to pathophysiological reasoning than to epidemiology [12]. The American Academy of Pediatrics made recommendations in 1992 [13] but official advice did not come until 1994 [14].

Gilbert el al show that the sequence the Netherlands > Britain > US (considering only these 3 countries) in new advice to put babies to sleep on their backs was paralleled by a similar sequence for changes in parental behaviour (as indicated by the sleeping positions of controls in case control studies during this period) and in the onsets of SIDS mortality declines. Noticeable declines in SIDS mortality in the US did not begin until 1992 [13].

Reference List

1. De AD, Day NE, Gore SM, Gilks WR, McGee MA: **AIDS: the statistical basis for public health.** *Stat Methods Med Res* 1993, **2:** 75-91.

2. Acheson ED: **The last convoy to Gorazde?** *Lancet* 1993, **342:** 43-44.

3. Landesman SH, Vieira J: **Acquired immune deficiency syndrome (AIDS). A review.** *Arch Intern Med* 1983, **143:** 2307-2309.

4. Golubjatnikov R, Pfister J, Tillotson T: **Homosexual promiscuity and the fear of AIDS.** *Lancet* 1983, **2:** 681.

5. Weller IV, Hindley DJ, Adler MW, Meldrum JT: **Gonorrhoea in homosexual men and media coverage of the acquired immune deficiency syndrome in London 1982-3.** *Br Med J (Clin Res Ed)* 1984, **289:** 1041.

6. Mihill C: **Babies should sleep on backs to cut deaths, says campaign**. The Times. 11-12-1991.

7. Gilbert R, Salanti G, Harden M, See S: **Infant sleeping position and the sudden infant death syndrome: systematic review of observational studies and historical review of recommendations from 1940 to 2002.** *Int J Epidemiol* 2005, **34:** 874-887.

8. Davies DP: **Cot death in Hong Kong: a rare problem?** *Lancet* 1985, **2:** 1346-1349.

9. Beal S: **Sleeping position and SIDS.** *Lancet* 1988, **2:** 512.

10. McKee M, Fulop N, Bouvier P, Hort A, Brand H, Rasmussen F *et al*.: **Preventing sudden infant deaths--the slow diffusion of an idea.** *Health Policy* 1996, **37:** 117-135.

11. **European Society for the study and prevention of infant death 13.** 1991.

12. Dwyer T, Ponsonby AL: **The decline of SIDS: a success story for epidemiology.** *Epidemiology* 1996, **7:** 323-325.

13. **American Academy of Pediatrics AAP Task Force on Infant Positioning and SIDS: Positioning and SIDS.** *Pediatrics* 1992, **89:** 1120-1126.

14. Willinger M, Hoffman HJ, Hartford RB: **Infant sleep position and risk for sudden infant death syndrome: report of meeting held January 13 and 14, 1994, National Institutes of Health, Bethesda, MD.** *Pediatrics* 1994, **93:** 814-819.
