# Supplementary material for: Informal knowledge transfer in the period before formal health education programmes: case studies of mass media coverage of HIV and SIDS in England and Wales
Source: BMC Public Health. 2007 Oct 17;7:293. doi: 10.1186/1471-2458-7-293 (PMC2194775; doi:10.1186/1471-2458-7-293)
Supplement: Additional file 2 — Additional Table 1: Brief chronology of scientific discoveries and publications in HIV/AIDS. From first description of the syndrome in March, 1981 to the licencing of blood tests in 1985. [file 1471-2458-7-293-S2.doc]

**Additional Table 1:** Brief chronology of scientific discoveries/ publications in HIV/AIDS

| Author, month, year published | Scientific advancement |
| --- | --- |
| Friedman-Kien et al, March, 1981[1] | Spectrum of otherwise rare infections and cancer first reported among American homosexual men |
| Dubois et al, December, 1981 [2] | First UK case documented |
| Masur et al, December, 1981 [3] | Reports on intravenous drug users |
| CDC, June, 1982 [4] | Sexually transmissible agent hypothesis |
| MMWR, July, 1982 [5] | Haemophiliacs at risk group |
| Kher, July,1982 [6] | AIDS term defined by the US Centres for Disease Control |
| Harris et al, May,1983 [7] | Heterosexual transmission reported |
| Gallo & Montagnier, 1983-1985 (reported April,1987) [8] | Viral markers in blood (1983) and semen (1984) were isolated  HIV sequenced (1985) |
| Pear, January, 1985 [9] | First blood tests licensed in the USA |

Reference List

1. Friedman-Kien AE: **Disseminated Kaposi's sarcoma syndrome in young homosexual men.** *J Am Acad Dermatol* 1981, **5:** 468-471.

2. du Bois RM, Branthwaite MA, Mikhail JR, Batten JC: **Primary Pneumocystis carinii and cytomegalovirus infections.** *Lancet* 1981, **2:** 1339.

3. Masur H, Michelis MA, Greene JB, Onorato I, Stouwe RA, Holzman RS *et al*.: **An outbreak of community-acquired Pneumocystis carinii pneumonia: initial manifestation of cellular immune dysfunction.** *N Engl J Med* 1981, **305:** 1431-1438.

4. **A cluster of Kaposi's sarcoma and Pneumocystis carinii pneumonia among homosexual male residents of Los Angeles and Orange Counties, California.** *MMWR Morb Mortal Wkly Rep* 1982, **31:** 305-307.

5. **Pneumocystis carinii pneumonia among persons with hemophilia A.** *MMWR Morb Mortal Wkly Rep* 1982, **31:** 365-367.

6. Kher U: **A name for the plague**. The Times. 27-7-1982.

7. Harris C, Small CB, Klein RS, Friedland GH, Moll B, Emeson EE *et al*: **Immunodeficiency in female sexual partners of men with the acquired immunodeficiency syndrome.** *N Engl J Med* 1983, **308:** 1181-1184.

8. Gallo RC, Montagnier L: **The chronology of AIDS research.** *Nature* 1987, **326:** 435-436.

9. Pear P: **AIDS blood test to be available in 2 to 6 weeks.** The New York Times. 3-3-1985.
