# Supplementary material for: Informal knowledge transfer in the period before formal health education programmes: case studies of mass media coverage of HIV and SIDS in England and Wales
Source: BMC Public Health. 2007 Oct 17;7:293. doi: 10.1186/1471-2458-7-293 (PMC2194775; doi:10.1186/1471-2458-7-293)
Supplement: Additional file 3 — Additional Table 2: Chronology of scientific publications on the association between SIDS and sleeping position. Reports of epidemiological studies of SIDS from 1965 to 1991. [file 1471-2458-7-293-S3.doc]

**Additional Table 2:** Chronology of scientific publications on the association between SIDS and sleeping position

| Author, month, year published | Key findings | Reported in mass media |
| --- | --- | --- |
| Froggatt, 1970 [1] | Case-control study  Gave no guidance on which position was safest |  |
| Davies, December, 1985 [2] | Letter noting low incidence of SIDS in Hong Kong and speculating a link with prone sleeping position |  |
| Beal, August 1988 [3] | Meta-analysis of 9 previous studies found significantly less SIDS infants than controls slept supine. |  |
| McGlashan’s, 1988 [4] | Evidence from Tasmania recommending against prone positioning |  |
| De Jonge, March,1989 [5] | Case control study found significantly increased risk of SIDS if sleeping prone. | Guardian 14.6.1989 Heather Welford “Cradled in safety- letting a baby sleep on its back may avoid cot death” |
| Fleming, July, 1990 [6] | Population case-control study showed that overheating and prone position independently associated with increased risk of SIDS, particularly in infants aged more than 70 days | Guardian 13.7.1990 David Brindle “Research links overheating and duvets to cot death syndrome”  Times 13.7.1990 Thomas Prentice “cot death victims may have been too hot in bed” |
| Dwyer, May, 1991 [7] | Tazmanian group running a prospective study – the results of the concurrent case control study showed an increased risk of SIDS is slept prone. |  |

Reference List

1. Frogatt P:  **Epidemiological aspects of the Northern Ireland Study.** Seattle. 32-46.

2. Davies DP: **Cot death in Hong Kong: a rare problem?** *Lancet* 1985, **2:** 1346-1349.

3. Beal S: **Sleeping position and SIDS.** *Lancet* 1988, **2:** 512.

4. McGlashan ND: **Sudden infant deaths in Tasmania, 1980-1986: a seven year prospective study.** *Soc Sci Med* 1989, **29:** 1015-1026.

5. de Jonge GA, Engelberts AC, Koomen-Liefting AJ, Kostense PJ: **Cot death and prone sleeping position in The Netherlands.** *BMJ* 1989, **298:** 722.

6. Fleming PJ, Gilbert R, Azaz Y, Berry PJ, Rudd PT, Stewart A *et al*.: **Interaction between bedding and sleeping position in the sudden infant death syndrome: a population based case-control study.** *BMJ* 1990, **301:** 85-89.

7. Dwyer T, Ponsonby AL, Newman NM, Gibbons LE: **Prospective cohort study of prone sleeping position and sudden infant death syndrome.** *Lancet* 1991, **337:** 1244-1247.
