# Supplementary material for: Informal knowledge transfer in the period before formal health education programmes: case studies of mass media coverage of HIV and SIDS in England and Wales
Source: BMC Public Health. 2007 Oct 17;7:293. doi: 10.1186/1471-2458-7-293 (PMC2194775; doi:10.1186/1471-2458-7-293)
Supplement: Additional file 4 — Additional Table 3: Data abstraction form for HIV/AIDS articles. Data abstraction form for HIV/AIDS articles. [file 1471-2458-7-293-S4.doc]

**Additional Table 3:** Data abstraction form for HIV/AIDS articles

| **Date** |  |
| --- | --- |
| **Publication** |  |
| **Title/Headline** |  |
| **Page number** |  |
| **Column (cm)** |  |
| **Author** |  |
| **Section**  e.g. letter, health, news, commentary |  |
| **Location referred to in story**   1. UK 2. North America 3. Europe 4. Elsewhere |  |
| **Source of story**   1. Case study 2. BMJ 3. Lancet 4. Doctor 5. Researcher 6. WHO 7. Terrence Higgins Trust 8. Government 9. Other |  |
| **Risk groups described**   1. Homosexuals/Bisexuals 2. Haemophiliacs 3. Heterosexuals 4. Drug users |  |
| **Transmission routes discussed**   1. Blood 2. Semen 3. Other |  |
| **Transmission methods discussed**   1. Gay sex 2. Straight sex 3. Other close physical contact 4. Social contact 5. Contaminated blood transfusion 6. Contaminated factor VIII 7. Other |  |
| **Educational messages**   1. Reduce promiscuity 2. Practice safe sex 3. Gay men not to donate blood 4. Other |  |
| **AIDS word count** |  |
| **Controversy**   1. Over reported research 2. High risk groups 3. Transmission routes 4. Donated blood contamination 5. Public / Media hysteria 6. Epidemic status |  |
| **Death Toll/Cases**   1. UK 2. USA 3. Elsewhere |  |
| **Frame**   1. Case study 2. Medical / Research 3. Public Health 4. Policy 5. Political |  |
| **Issues**   - Risk of epidemic/to population - Haemophiliac dangers/sympathy - Blood donation - High profile deaths - Social isolation of carriers - Uncertainty - Gay labels: eg GRID, gay plague, etc - Associated diseases - Testing individuals - Human suffering - Fears - Changing working practice/habits - Initiatives: Research/support - High mortality/No cure - Discounted theories - Source of AIDS - Immune system collapse - Opinions |  |
