# Supplementary material for: Informal knowledge transfer in the period before formal health education programmes: case studies of mass media coverage of HIV and SIDS in England and Wales
Source: BMC Public Health. 2007 Oct 17;7:293. doi: 10.1186/1471-2458-7-293 (PMC2194775; doi:10.1186/1471-2458-7-293)
Supplement: Additional file 5 — Additional Table 4: Data abstraction form for SIDS articles. Data abstraction form for SIDS articles. [file 1471-2458-7-293-S5.doc]

**Additional Table 4:** Data abstraction form for SIDS articles

| **Date** |  |
| --- | --- |
| **Source** |  |
| **Length** |  |
| **Headline** (e.g. mentions sleeping position) |  |
| **Section / by line** (e.g. Medical / science) |  |
| Is sleeping position the main topic of the article? |  |
| **Frame**   - Research - Public Health - Personal - Parenting |  |
| **Theme**   - Suggestion only that position may be a factor - More SIDS babies found in prone position - Increased risk of SIDS in prone position - Quoting studies - Quoting experts - Professional opinion is divided - Parents are confused   If supine may chock on vomit  If on side may roll forward   - Other factors influence SIDS - A clear recommendation is made   By: |  |
| **Overall argument is for: side/prone/supine/equivocal** |  |
